# The Use of Tractography-Based Targeting in Deep Brain Stimulation for Psychiatric Indications

**DOI:** 10.3389/fnhum.2020.588423

**Published:** 2020-11-16

**Authors:** Benjamin Davidson, Nir Lipsman, Ying Meng, Jennifer S. Rabin, Peter Giacobbe, Clement Hamani

**Affiliations:** ^1^Sunnybrook Research Institute, Toronto, ON, Canada; ^2^Harquail Centre for Neuromodulation, Sunnybrook Health Sciences Centre, Toronto, ON, Canada; ^3^Division of Neurosurgery, Sunnybrook Health Sciences Centre, University of Toronto, Toronto, ON, Canada; ^4^Division of Neurology, Department of Medicine, Sunnybrook Health Sciences Centre, University of Toronto, Toronto, ON, Canada; ^5^Department of Psychiatry, Sunnybrook Health Sciences Centre, University of Toronto, Toronto, ON, Canada

**Keywords:** deep brain stimulation, depression, obsessive compulsive disorder, post-traumatic stress disorder, diffusion tensor imaging, tractography

## Abstract

Deep Brain Stimulation (DBS) has been investigated as a treatment option for patients with refractory psychiatric illness. Over the past two decades, neuroimaging developments have helped to advance the field, particularly the use of diffusion tensor imaging (DTI) and tractographic reconstruction of white-matter pathways. In this article, we review translational considerations and how DTI and tractography have been used to improve targeting during DBS surgery for depression, obsessive compulsive disorder (OCD) and post-traumatic stress disorder (PTSD).

## Introduction

Psychiatric illness remains among the leading causes of disability worldwide (World Health Organization, 2017). Common conditions such as major depressive disorder (MDD) and obsessive compulsive disorder (OCD) are resistant to guideline-concordant pharmacotherapy and psychotherapy in up to one third of cases (Trivedi et al., 2006; Hirschtritt et al., 2017). Patients with treatment resistant psychiatric illness have a significantly increased usage of healthcare resources and risk of suicide (Kessler, 2012; World Health Organization, 2017). The high prevalence and often fatal prognosis of refractory psychiatric illnesses emphasizes the need to develop novel treatment options for this patient population.

Psychiatric surgery, namely deep brain stimulation (DBS) or ablative neurosurgery, is an important treatment option for patients with refractory psychiatric illness. DBS involves the surgical placement of electrodes in the brain, which deliver continuous low-level stimulation to a precisely targeted node (Awan et al., 2009; Hamani and Nobrega, 2010, 2012; Hamani et al., 2010). Ablative neurosurgery, involves the creation of a focal lesion in the brain—performed either with surgery (Christmas et al., 2011), stereotactic radiosurgery (Rasmussen et al., 2018), or magnetic resonance guided focused ultrasound (MRgFUS) (Kim et al., 2018; Davidson et al., 2020b).

Over the past two decades, the field of neuroimaging has evolved considerably. Advanced magnetic resonance imaging (MRI) sequences such as echo-planar imaging, used for functional MRI (fMRI), and diffusion tensor imaging (DTI), used for the tractographic reconstruction of white-matter pathways, has added a dizzying array of possibility, but also complexity, to performing psychiatric neurosurgery. In 2013, Schlaepfer et al. reported the use of DTI to target a structure termed the “superolateral branch of the medial forebrain bundle” (slMFB) (Schlaepfer et al., 2013), which has since been renamed the ventral tegmental area projection pathway (VTApp) (Coenen et al., 2020). Since then, there has been extensive translational work, resulting in improved outcomes (Riva-Posse et al., 2018) and the emergence of a new era of circuit-based neurosurgery (Boutet et al., 2019; Horn, 2019).

In this article, we review the use of advanced neuroimaging techniques in psychiatric DBS, particularly highlighting the methods which have been translated into clinical practice. This review will be divided based on the major targets currently used for psychiatric surgery: subcallosal cingulum (SCC), VTApp, ventral capsule/ventral striatum (VC/VS).

## Ventral Tegmental Area Projection Pathway

The first translation of advanced neuroimaging techniques to psychiatric surgery in humans was in the stimulation of the VTApp in the treatment of MDD (Schlaepfer et al., 2013). The VTApp was originally selected as a potential target following the observation of hypomania developing in a patient with Parkinson’s disease who received subthalamic nucleus (STN) DBS, where the active contact was located too medially (Coenen et al., 2009). Though originally referred to as the slMFB, several groups have suggested that this tract may in fact be a hyper-direct connection from the prefrontal cortex to the anteromedial STN. For the purposes of this article, we will refer to this tract as the ventral tegmental projection pathway.

**FIGURE 1:**
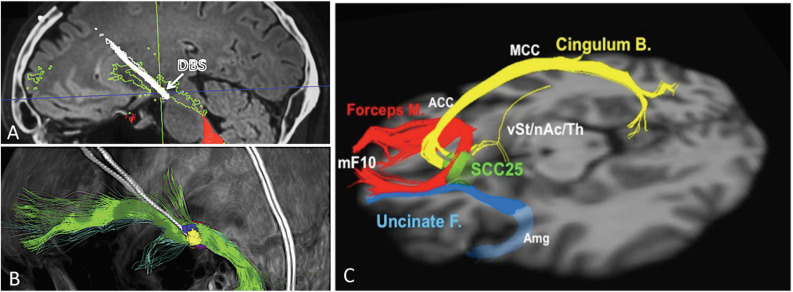
Ventral tegmental area projection pathway (VTApp) DBS. **(A)** Outline shows how DBS electrode traverses the VTApp, formerly suggested to be the superolateral branch of the medial forebrain bundle (green). **(B)** Three-dimensional view from lateral and left. **(C)** Optimal SCC DBS Fiber Bundle Target Template. Red: Forceps Minor, Blue: Uncinate Fasciculus, Yellow: Cingulate Bundle. Abbreviations: mF10, medial frontal (Brodmann Area 10); Forceps M., forceps minor; Uncinate F., uncinate fasciculus; Cingulum B., cingulum bundle; vSt, ventral Striatum; nAc, nucleus accumbens; Th, thalamus; SCC25, subcallosal cingulate cortex (BA25); Amg, amygdala; ACC, anterior cingulate cortex; MCC, middle cingulate cortex. Adapted and reprinted from Coenen et al. (2018), with permission from Elsevier. Reprinted from Riva-Posse et al. (2014), with permission from Elsevier.

The VTApp is consistently found within a region recently coined as the “therapeutic triangle,” located immediately lateral to the ventral tegmental area (Coenen et al., 2018). The therapeutic triangle is defined anteriorly by the mamillary body, posteromedially by the red nucleus, and posterolaterally by the substantia nigra/STN. Since the VTApp cannot be appreciated on standard structural MRI sequences, deterministic DTI is used to optimize the electrode depth and trajectory, so as to maximize contact with the VTApp. Due to the close proximity of the oculomotor nerve, the acute stimulation effect of diplopia and dizziness serves as a confirmation of accurate electrode placement, but also limits the amplitude of stimulation. Figures 1A,B depicts an electrode placed in the region of the VTApp (Coenen et al., 2018).

In the first open-label trial of VTApp DBS (FORSEE I; FORebrain Simulation dEprEssion), clinical response and remission were achieved in 6/7 and 4/7 patients, respectively (Schlaepfer et al., 2013). Particularly impressive was the fact that all 7 patients experienced acute intraoperative appetitive responses, and 5/7 patients achieved responder status by 1 week post-operatively. Such a rapid rate of improvement was unprecedented in the psychiatric DBS field, even amongst open-label studies. At long-term follow-up, these improvements were maintained for at least 4 years (Bewernick et al., 2017). In a second open-label series of VTApp DBS from the same group (FORSEE II), there was a nearly 50% reduction in the mean depression ratings as early as 1 week, and a 100% response rate at 12 months (Coenen et al., 2019).

The approach to targeting the VTApp reported in the FORESEE trials, has been implemented by at least two other centers, with one reporting promising results (Fenoy et al., 2018). The other center reported a lack of robust intraoperative findings or postoperative response among two anhedonic MDD patients despite following a similar DTI-based targeting approach (Davidson et al., 2020a). Although the VTApp remains an appealing target for DBS in the treatment of refractory MDD, especially given its reported rapid clinical response rates, more nuanced patient or target selection may be required; different targets may offer better clinical effect depending on depression subtypes, neuroimaging biomarkers, or the results of intraoperative stimulation (Widge et al., 2018). For example, given the high rate of intraoperative appetitive responses among those patients who obtain clinical benefit with VTApp DBS, it could be hypothesized that in patients where appetitive responses are not observed, an alternative target should be stimulated. It is also important to acknowledge that all VTApp data to date has come from open-label studies, and although promising, a large randomized trial is still needed (Coenen et al., 2019).

## Subcallosal Cingulum

The SCC target was first selected based on its critical role in the network involved in the modulation of negative affect (Seminowicz et al., 2004; Mayberg et al., 2005). Despite promising open-label data, an industry-sponsored randomized controlled trial (RCT) failed to show a difference between active and sham stimulation at 6 months (Holtzheimer et al., 2017). Connectivity-based studies have suggested that DTI-based targeting, may be one of many critical factors needed to demonstrate efficacy (Mayberg et al., 2016; Widge et al., 2016).

In the first published series, DBS electrodes were implanted at the transition between gray and white matter beneath the genu of the corpus callosum (Hamani et al., 2011). Based upon anatomic positioning of the active contacts, there was no appreciable difference in location between responders and non-responders (Hamani et al., 2011). On subsequent analyses, it soon became apparent that the therapeutic benefit was associated with the pattern of axonal white-matter tracts stimulated, rather than the location within the gray matter (Riva-Posse et al., 2014).

In a proof of concept analysis, the white matter tracts stimulated by SCC DBS electrodes were mapped using probabilistic DTI in a single subject who responded to treatment (Lujan et al., 2013). On both the left and right side, the therapeutic contact was positioned at the intersection of the cingulum, forceps minor, and frontostriatal projections. Importantly, the most ventral contacts, located at the gray–white junction, did not intersect with this white matter blueprint, and only contacted frontostriatal fibers. Based on these results, a larger retrospective analysis in patients who had received open-label SCC-DBS demonstrated a shared connectome blueprint amongst responders than was not seen in non-responders (Riva-Posse et al., 2014). In this study, the strongest response was observed in patients where the volume of tissue activated (VTA) was situated at the intersection of the forceps minor, cingulum bundle, frontostriatal projections, as well as the uncinate fasciculus (UF) (Figure 1C). When this white-matter blueprint targeting strategy was used prospectively in an open-label study, the 6-month response rate improved from 41 to 73% (Riva-Posse et al., 2018). The acute autonomic effects of SCC DBS observed intraoperatively appear to be directly correlated to the degree of structural connectivity between the VTA and the mid-cingulate cortex (via the cingulum bundle) (Riva-Posse et al., 2019).

The white-matter blueprint SCC targeting scheme is now being used by other groups in trials of SCC-DBS (Hamani et al., 2020; Ramasubbu et al., 2020), demonstrating the translational impact of this work. One group has attempted to disentangle the contribution of each of the four tracts in this blueprint toward an eventual clinical response (Clark et al., 2020). Using data from an open-label study, they reported that excessive stimulation of the forceps minor (especially its dorsal component) is associated with non-response, while stimulation of the UF is associated with clinical improvement (Clark et al., 2020). These results are not necessarily contradictory to the findings of Riva-Posse and colleagues, who have yet to report each individual tract’s association with clinical response. It should also be noted that these two groups use different methods for predicting the size of the VTA (Butson et al., 2007; Chaturvedi et al., 2013), leading to dramatic differences in activated volume. The use of open-source VTA-modeling software, such as that provided by the Lead-DBS software might help with the generalizability of these studies (Horn et al., 2019).

Recently, our group extended this DTI-based SCC targeting approach to the treatment of refractory post-traumatic stress disorder (PTSD) (Hamani et al., 2020). PTSD, which develops as a maladaptive response to previous traumatic events, is characterized by hypervigilance, frequent “re-experiencing” of traumatic events, and dissociation/depersonalization. There is a high rate of comorbid depression and anxiety (Kessler et al., 2005). Preclinical and human studies of PTSD have demonstrated hyperactivity in the amygdala, which is likely due to inadequate top-down inhibition from an underactive ventromedial prefrontal cortex (Milad et al., 2009; Reznikov et al., 2015). In a rodent model, DBS delivered to the infralimbic cortex (considered to be homologous to the rodent homolog of the SCC) (Hamani et al., 2014a; Reznikov et al., 2016) improved fear-extinction and reduced anxiety-type behavior while decreasing cell-firing of principal cells in the basolateral amygdala (Reznikov et al., 2018).

**FIGURE 2:**
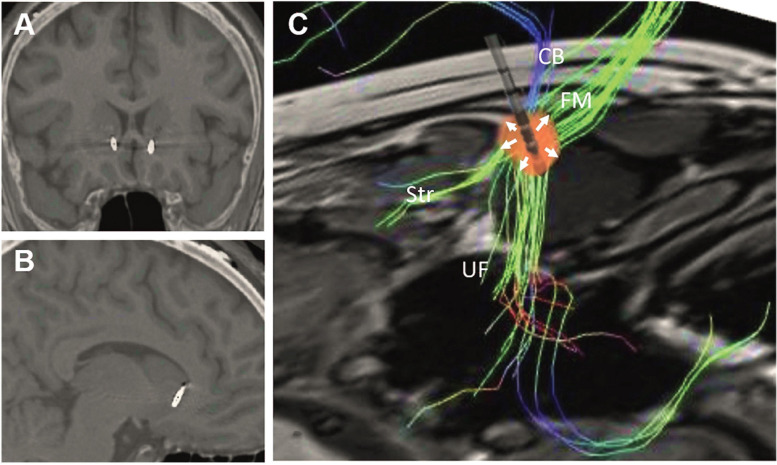
Postoperative computed tomography fused with preoperative magnetic resonance images showing the location of the electrodes in the **(A)** coronal and **(B)** sagittal planes. **(C)** Reconstruction of preoperative tractography and schematic representation of an implanted electrode. The presented spread of current (red spherical shape) was adapted to reflect the fact that 67% of the current was delivered medially through contacts in third ring (arrows pointing upward), while 33% spread laterally though a lateral contact in the second ring (arrows pointing downward). Under these circumstances, stimulation through the second ring would largely affect the uncinate fasciculus (UF), whereas stimulation of the third ring would largely modulate the cingulate bundle (CB), forceps minor (FM), and frontostriatal (Str) projections. Reprinted from Hamani et al. (2020), with permission from Elsevier.

Based on these results, we hypothesized that the DTI-informed white matter SCC target could be beneficial in the treatment of PTSD, partly through stimulation of UF fibers passing from the prefrontal cortex to the amygdala, and partly through the modulation of the affective network through the cingulum and forceps minor. In order to maximize stimulation of the UF and the cingulum bundle, we used directional DBS, with current directed toward the uncinate fasciulus as well as to the fiber blueprint proposed by Riva-Posse et al. (2014) (Figure 2). In a open-label proof-of-concept index case, this directional stimulation approach led to a dramatic and robust reduction in PTSD symptoms (Hamani et al., 2020). As additional centers begin to apply this DTI-informed targeting of the SCC, it will likely be possible to further optimize this approach, potentially associating improvement in specific symptom subtypes with individual tracts of the blueprint.

Recently two articles have suggested potential imaging-based biomarkers of response to SCC-DBS. In the first study, a voxel-based morphometry analysis of 27 patients with SCC DBS suggested that a larger preoperative SCC volume is associated with eventual clinical response (Sankar et al., 2020). Another center reported that preoperative SCC hypermetabolism may predict responder status (Brown et al., 2020). Although there is not yet sufficient evidence to use these biomarkers to select patients, the field seems to be moving toward imaging-based patient and target selection.

## Ventral Striatum/Ventral Capsule

The ventral striatum/ventral capsule (VC/VS), used here to refer to the highly similar ventral anterior limb of the internal capsule (vALIC) and the nucleus accumbens (NAc) targets, is the most common DBS target in the treatment of OCD (Hamani et al., 2014b; Denys et al., 2020), but also frequently used in the treatment of MDD (Dougherty et al., 2015). Although open-label trials of VC/VS DBS have yielded long-term response rates ranging from 40 to 66% for OCD and MDD (Greenberg et al., 2010; Bewernick et al., 2012; van der Wal et al., 2019; Winter et al., 2020), an industry-sponsored RCT of VC/VS DBS for MDD failed to show a difference between true and placebo stimulation for MDD (Dougherty et al., 2015). An analogous RCT has not been published with VC/VS DBS for OCD, though class I evidence has been obtained from an RCT showing that STN DBS was better than sham stimulation (Mallet et al., 2008). VC/VS DBS studies in which patients underwent a blinded active vs. sham stimulation phase following long-term optimization have shown a positive outcome for both OCD (Denys et al., 2010) and depression (Bergfeld et al., 2016). The use of DBS for OCD is approved in many countries, some under humanitarian-device exemption.

VC/VS DBS was originally implemented as means of mimicking the effect of stereotactic lesioning (Nuttin et al., 1999), a procedure known as anterior capsulotomy, which has been performed since 1949 (Talairach et al., 1949). Over the years, the VC/VS target has migrated posteriorly, almost to the level of the anterior commissure, based solely on clinical experience of better outcomes associated with more posterior stimulation (Greenberg et al., 2010; Luyten et al., 2016; Raymaekers et al., 2017). To our knowledge, there has not yet been prospective use of advanced imaging techniques, such as DTI or fMRI, for targeting within the VC/VS, although this is likely to change given the multitude of recent studies delineating the anatomical nuances of this region (Hartmann et al., 2015; Avecillas-Chasin et al., 2019; Coenen et al., 2020).

Currently, VC/VS DBS or vALIC ablation is targeted based on standardized measurements relative to the anterior commissure and the midline, despite there being numerous distinct projection bundles found in the vicinity. These white matter projections are organized along a ventral-dorsal and medial-lateral gradient. Within the ALIC, fibers located ventromedially are more likely to project to ventromedial cortical targets, such as the ventromedial prefrontal cortex, whereas fibers found dorsolaterally project to targets such as the dorsolateral prefrontal cortex (Lehman et al., 2011; Avecillas-Chasin et al., 2019). Despite this consistent topographic organization, there is substantial inter-individual variability, as well as “interweaving” of fibers within the ALIC (Makris et al., 2016; Nanda et al., 2017).

Three recent studies have suggested that the VTApp within ALIC (which may represents the hyperdirect cortical-STN connection), may be a critical fiber tract leading to clinical response in DBS for OCD (Baldermann et al., 2019; Liebrand et al., 2019; Li et al., 2020). ALIC fibers associated with a good outcome have been postulated to run dorsal to the NAc, near the bed nucleus of the stria terminalis, entering the ventral part of the thalamus at the border of the anterior and inferior thalamic peduncle (Baldermann et al., 2019). These ultimately connect the prefrontal cortex with the medial dorsal nucleus of the thalamus and the STN (Baldermann et al., 2019). Furthermore, connectivity to the medial frontal gyrus may mediate antidepressant effects (Baldermann et al., 2019).

Another approach is to categorize the tracts on the ALIC based on their involvement in specific circuits, with reward and affect circuits being found more ventrally, and cognitive control/decision making circuits being located more dorsally (Coenen et al., 2020). As DBS and ablative procedures are both theorized to function by interrupting pathological circuit-based oscillations, targeting could be adjusted in the ventral-dorsal or medial lateral direction based on a patient’s symptom profile. For example—in OCD patients with especially prominent symptoms of cognitive inflexibility, a more dorsally activated contact (or lesion) may be optimal, whereas patients with more prominent mood symptoms might benefit from ventral targeting (Coenen et al., 2020).

## Individualized vs. Normative Imaging

As neuroimaging techniques evolve to the point of translation into clinical care, a dilemma has arisen as to the comparative value of individualized DTI and fMRI data, vs. large normative “averaged” datasets. The advantages of large normative datasets include an improved signal-to-noise ratio (SNR), potential use of state-of-the art equipment (i.e., Human Connectome Project) (Van Essen et al., 2013), and allowing for a more universal scientific language and comparison between centers (Horn and Blankenburg, 2016). However, given the considerable inter-individual variability in brain structure and connectivity, applying normative imaging at the patient level might prevent the ability to “personalize” neuromodulation treatments (Fox et al., 2013; Cash et al., 2020). For instance, some authors have suggested that the inter-individual variability of tract positioning within the ALIC will require subject-specific high-resolution DTI in order to personalize targets (Makris et al., 2016; Nanda et al., 2017). On the other hand, single-subject DTI can introduce substantial variance, and according some authors, may not be ready for mainstream use in targeting (Jakab et al., 2016; Petersen et al., 2017).

In a small double-blinded sham-stimulation controlled series of VC/VS implanted OCD patients, it was demonstrated that individualized fMRI/DTI could be used to determine optimal electrode contacts (Barcia et al., 2019). Patients underwent a symptom provocation task during fMRI scanning, revealing distinct areas of cortical activation based on their predominant compulsion symptoms (i.e., contamination obsessions activate a different cortical region than checking obsessions). Among responders, the most effective contacts could be distinguished by their connectivity to the cortical region displaying activation on the symptom-provocation fMRI. In some patients, this involved a more dorsal contact, while in others more ventral contacts proved most effective. This suggests that prospectively, patients could be programed based on their individual fMRI pattern of prefrontal activation during symptom provocation. Although these findings are preliminary, they emphasize the need to continue developing patient-specific advanced neuroimaging methods, despite the challenges of low SNR and high variability.

In contrast, emphasizing the advantage of large normative datasets, a recent study used DTI data derived from the HCP, to identify a common tract distinguishing responders from non-responders following VC/VS DBS for OCD (Li et al., 2020). The tract is part of a hyperdirect circuit, projecting from the anterior cingulate and lateral prefrontal cortex to the anteromedial STN. Their analysis included patients from four different centers, with DBS implanted at the VC/VS or STN. In patients with VC/VS DBS, non-responders tended to have a VTA placed too ventrally, with the critical tract passing above. In patients with STN DBS, a VTA located too dorsally resulted in the tract passing below, and a reduced likelihood of clinical response. Through the use of several cohorts, they were able to demonstrate significant out-of-sample predictive capabilities of this tract, suggesting a unified mechanism underlying clinical response to both STN and ALIC DBS in refractory OCD. It should be noted that this multi-centered data-sharing effort used a mixture of open-label and blinded clinical data.

Currently, the only prospective implementation of DTI for psychiatric surgery involves patient-level data, for SCC and VTApp stimulation. It has yet to be assessed if and how these targets would be affected by using normative data. Normative datasets have to be non-linearly warped into patient-space, which can introduce an additional source of error, and may erode some of the advantages of higher-resolution imaging.

## Correlating Imaging Findings With Outcomes

Many of the neuroimaging advances seen in psychiatric surgery are translated from the field of movement disorders (Horn et al., 2017). Application in psychiatric surgery, however, is complicated by two central factors. Firstly, outcome measurement is much more challenging in psychiatric disorders than movement disorders, where clinical improvements are often immediate and easily quantified (i.e., the Unified Parkinson’s Disease Rating Scale). Following psychiatric surgery, clinical results often take months to manifest, and even then, there is considerable debate over the optimal way to measure outcomes (Rabin et al., 2020). Although the outcome of most psychiatric surgery trials is distilled down to a single clinical score, psychiatric illness may not be accurately characterized in such a manner. To some degree, the success of neuroimaging analyses in psychiatric surgery is related to the validity of outcome scores. As neuroimaging analyses become increasingly sophisticated, it will be crucial for measurement of psychiatric outcomes to similarly become more nuanced. Secondly, the amount of data available for analysis is often limited, due to factors including limited funding, lack of access, and a reluctance to refer patients for psychiatric surgery (Mendelsohn et al., 2013; Cormier et al., 2019). Multi-centered data-sharing efforts, such as the recent study by Li et al. (2020), are increasingly becoming a necessity.

## Conclusion

Advanced neuroimaging techniques have now begun to influence the clinical practice of psychiatric neurosurgery. DTI targeting methods are being used routinely in SCC and VTApp DBS for MDD. Although VC/VS targeting is still performed with conventional targeting based on structural MRI, the findings of several recent DTI and fMRI studies have suggested methods for improved targeting. There continues to be a role for the use of both patient-specific imaging and large normative datasets, with both offering distinct advantages. As an added wrinkle of complexity, the clinical scores upon which imaging analyses are based, are often not well represented by a single number, and future imaging studies will need to develop more advances ways of accommodating clinical heterogeneity. With advanced neuroimaging having already been translated into human clinical trials, the future of neuroimaging in psychiatric surgery is very promising.

## Author Contributions

BD, YM, and CH wrote the manuscript. NL, JR, and PG provided insightful comments and reviewed content. All authors contributed to the article and approved the submitted version.

## Conflict of Interest

CH was part of an unrelated advisory board for Medtronic. PG reports personal fees from Janssen, non-financial support from St. Jude Medical, outside the submitted work. The remaining authors declare that the research was conducted in the absence of any commercial or financial relationships that could be construed as a potential conflict of interest.

## References

[B1] Avecillas-Chasin J. M. Hurwitz T. A. Bogod N. M. Honey C. R. (2019). An analysis of clinical outcome and tractography following bilateral anterior capsulotomy for depression. *Stereotact. Funct. Neurosurg.* 97 5–6. 10.1159/000505077 31865344

[B2] Awan N. R. Lozano A. Hamani C. (2009). Deep brain stimulation: current and future perspectives. *Neurosurg. Focus.* 27:E2. 10.3171/2009.4.FOCUS0982 19569890

[B3] Baldermann J. C. Melzer C. Zapf A. Kohl S. Timmermann L. Tittgemeyer M. (2019). Connectivity profile predictive of effective deep brain stimulation in obsessive-compulsive disorder. *Biol. Psychiatry*. 85 735–743. 10.1016/j.biopsych.2018.12.019 30777287

[B4] Barcia J. A. Avecillas-Chasin J. M. Nombela C. Arza R. Garcia-Albea J. Pineda-Pardo J. A. (2019). Personalized striatal targets for deep brain stimulation in obsessive-compulsive disorder. *Brain Stimul.* 12 724–734. 10.1016/j.brs.2018.12.226 30670359

[B5] Bergfeld I. O. Mantione M. Hoogendoorn M. L. Ruhe H. G. Notten P. Van Laarhoven J. (2016). Deep brain stimulation of the ventral anterior limb of the internal capsule for treatment-resistant depression: a randomized clinical trial. *JAMA Psychiatr.* 73 456–464. 10.1001/jamapsychiatry.2016.0152 27049915

[B6] Bewernick B. H. Kayser S. Gippert S. M. Switala C. Coenen V. A. Schlaepfer T. E. (2017). Deep brain stimulation to the medial forebrain bundle for depression- long-term outcomes and a novel data analysis strategy. *Brain Stimul.* 10 664–671. 10.1016/j.brs.2017.01.581 28259544

[B7] Bewernick B. H. Kayser S. Sturm V. Schlaepfer T. E. (2012). Long-term effects of nucleus accumbens deep brain stimulation in treatment-resistant depression: evidence for sustained efficacy. *Neuropsychopharmacology* 37 1975–1985. 10.1038/npp.2012.44 22473055 PMC3398749

[B8] Boutet A. Gramer R. Steele C. J. Elias G. J. B. Germann J. Maciel R. (2019). Neuroimaging technological advancements for targeting in functional neurosurgery. *Curr. Neurol. Neurosci. Rep.* 19:42. 10.1007/s11910-019-0961-8 31144155

[B9] Brown E. C. Clark D. L. Forkert N. D. Molnar C. P. Kiss Z. H. T. Ramasubbu R. (2020). Metabolic activity in subcallosal cingulate predicts response to deep brain stimulation for depression. *Neuropsychopharmacology* 45 1681–1688. 10.1038/s41386-020-0745-5 32580207 PMC7419290

[B10] Butson C. R. Cooper S. E. Henderson J. M. Mcintyre C. C. (2007). Patient-specific analysis of the volume of tissue activated during deep brain stimulation. *Neuroimage* 34 661–670. 10.1016/j.neuroimage.2006.09.034 17113789 PMC1794656

[B11] Cash R. F. C. Weigand A. Zalesky A. Siddiqi S. H. Downar J. Fitzgerald P. B. (2020). Using brain imaging to improve spatial targeting of TMS for depression. *Biol. Psychiatry* 7:S0006-3223(20)31668-1 [Epub ahead of print]. 10.1016/j.biopsych.2020.05.033 32800379

[B12] Chaturvedi A. Lujan J. L. Mcintyre C. C. (2013). Artificial neural network based characterization of the volume of tissue activated during deep brain stimulation. *J. Neural Eng.* 10:056023 10.1088/1741-2560/10/5/056023 PMC4115460 24060691

[B13] Christmas D. Eljamel M. S. Butler S. Hazari H. Macvicar R. Steele J. D. (2011). Long term outcome of thermal anterior capsulotomy for chronic, treatment refractory depression. *J. Neurol. Neurosurg. Psychiatry* 82 594–600. 10.1136/jnnp.2010.217901 21172856

[B14] Clark D. L. Johnson K. A. Butson C. R. Lebel C. Gobbi D. Ramasubbu R. (2020). Tract-based analysis of target engagement by subcallosal cingulate deep brain stimulation for treatment resistant depression. *Brain Stimul.* 13 1094–1101. 10.1016/j.brs.2020.03.006 32417668

[B15] Coenen V. Schlaepfer T. Sajonz B. Dobrossy M. Kaller C. P. Urbach H. (2020). Tractographic description of major subcortical projection pathways passing the anterior limb of the internal capsule. Corticopetal organization of networks relevant for psychiatric disorders. *Neuroimage Clin.* 25:102165. 10.1016/j.nicl.2020.102165 31954987 PMC6965747

[B16] Coenen V. A. Bewernick B. H. Kayser S. Kilian H. Bostrom J. Greschus S. (2019). Superolateral medial forebrain bundle deep brain stimulation in major depression: a gateway trial. *Neuropsychopharmacology* 44 1224–1232. 10.1038/s41386-019-0369-9 30867553 PMC6785007

[B17] Coenen V. A. Honey C. R. Hurwitz T. Rahman A. A. Mcmaster J. Burgel U. (2009). Medial forebrain bundle stimulation as a pathophysiological mechanism for hypomania in subthalamic nucleus deep brain stimulation for Parkinson’s disease. *Neurosurgery* 64 1106–1114. 10.1227/01.neu.0000345631.54446.06 19487890

[B18] Coenen V. A. Sajonz B. Reisert M. Bostroem J. Bewernick B. Urbach H. (2018). Tractography-assisted deep brain stimulation of the superolateral branch of the medial forebrain bundle (slMFB DBS) in major depression. *Neuroimage Clin.* 20 580–593. 10.1016/j.nicl.2018.08.020 30186762 PMC6120598

[B19] Cormier J. Iorio-Morin C. Mathieu D. Ducharme S. (2019). Psychiatric neurosurgery: a survey on the perceptions of psychiatrists and residents. *Can. J. Neurol. Sci.* 46 303–310. 10.1017/cjn.2019.5 30975240

[B20] Davidson B. Giacobbe P. Mithani K. Levitt A. Rabin J. S. Lipsman N. (2020a). Lack of clinical response to deep brain stimulation of the medial forebrain bundle in depression. *Brain Stimul.* 13 1268–1270. 10.1016/j.brs.2020.06.010 32540453

[B21] Davidson B. Hamani C. Rabin J. S. Goubran M. Meng Y. Huang Y. (2020b). Magnetic resonance-guided focused ultrasound capsulotomy for refractory obsessive compulsive disorder and major depressive disorder: clinical and imaging results from two phase I trials. *Mol. Psychiatry* 25 1–12. 10.1038/s41380-020-0737-1 32404942

[B22] Denys D. Graat I. Mocking R. De Koning P. Vulink N. Figee M. (2020). Efficacy of deep brain stimulation of the ventral anterior limb of the internal capsule for refractory obsessive-compulsive disorder: a clinical cohort of 70 patients. *Am. J. Psychiatry* 177 265–271. 10.1176/appi.ajp.2019.19060656 31906709

[B23] Denys D. Mantione M. Figee M. Van Den Munckhof P. Koerselman F. Westenberg H. (2010). Deep brain stimulation of the nucleus accumbens for treatment-refractory obsessive-compulsive disorder. *Arch. Gen. Psychiatry* 67 1061–1068. 20921122 10.1001/archgenpsychiatry.2010.122

[B24] Dougherty D. D. Rezai A. R. Carpenter L. L. Howland R. H. Bhati M. T. O’reardon J. P. (2015). A randomized sham-controlled trial of deep brain stimulation of the ventral capsule/ventral striatum for chronic treatment-resistant depression. *Biol. Psychiatry* 78 240–248. 10.1016/j.biopsych.2014.11.023 25726497

[B25] Fenoy A. J. Schulz P. E. Selvaraj S. Burrows C. L. Zunta-Soares G. Durkin K. (2018). A longitudinal study on deep brain stimulation of the medial forebrain bundle for treatment-resistant depression. *Transl. Psychiatry* 8:111. 10.1038/s41398-018-0160-4 PMC5986795 29867109

[B26] Fox M. D. Liu H. Pascual-Leone A. (2013). Identification of reproducible individualized targets for treatment of depression with TMS based on intrinsic connectivity. *Neuroimage* 66 151–160. 10.1016/j.neuroimage.2012.10.082 23142067 PMC3594474

[B27] Greenberg B. D. Gabriels L. A. Malone D. A. Jr. Rezai A. R. Friehs G. M. (2010). Deep brain stimulation of the ventral internal capsule/ventral striatum for obsessive-compulsive disorder: worldwide experience. *Mol. Psychiatry* 15 64–79. 18490925 10.1038/mp.2008.55 PMC3790898

[B28] Hamani C. Amorim B. O. Wheeler A. L. Diwan M. Driesslein K. Covolan L. (2014a). Deep brain stimulation in rats: different targets induce similar antidepressant-like effects but influence different circuits. *Neurobiol. Dis.* 71 205–214. 10.1016/j.nbd.2014.08.007 25131446 PMC5756089

[B29] Hamani C. Pilitsis J. Rughani A. I. Rosenow J. M. Patil P. G. Slavin K. S. (2014b). Deep brain stimulation for obsessive-compulsive disorder: systematic review and evidence-based guideline sponsored by the American Society for Stereotactic and Functional Neurosurgery and the Congress of Neurological Surgeons (CNS) and endorsed by the CNS and american association of neurological surgeons. *Neurosurgery* 75 327–333. 25050579 10.1227/NEU.0000000000000499

[B30] Hamani C. Davidson B. Levitt A. Meng Y. Corchs F. Abrahao A. (2020). Deep Brain Stimulation for treatment resistant post-traumatic stress disorder: a feasibility study. *Biol. Psychiatry* 1:S0006-3223(20)31624-3 [Epub ahead of print].

[B31] Hamani C. Mayberg H. Stone S. Laxton A. Haber S. Lozano A. M. (2011). The subcallosal cingulate gyrus in the context of major depression. *Biol. Psychiatry* 69 301–308. 10.1016/j.biopsych.2010.09.034 21145043

[B32] Hamani C. Nobrega J. N. (2010). Deep brain stimulation in clinical trials and animal models of depression. *Eur. J. Neurosci.* 32 1109–1117. 10.1111/j.1460-9568.2010.07414.x 21039950

[B33] Hamani C. Nobrega J. N. (2012). Preclinical studies modeling deep brain stimulation for depression. *Biol. Psychiatry* 72 916–923. 10.1016/j.biopsych.2012.05.024 22748616 PMC5633367

[B34] Hamani C. Nobrega J. N. Lozano A. M. (2010). Deep brain stimulation in clinical practice and in animal models. *Clin. Pharmacol. Ther.* 88 559–562. 10.1038/clpt.2010.133 20720537

[B35] Hartmann C. J. Lujan J. L. Chaturvedi A. Goodman W. K. Okun M. S. Mcintyre C. C. (2015). Tractography activation patterns in dorsolateral prefrontal cortex suggest better clinical responses in OCD DBS. *Front. Neurosci.* 9:519. 10.3389/fnins.2015.00519 26834544 PMC4717315

[B36] Hirschtritt M. E. Bloch M. H. Mathews C. A. (2017). Obsessive-compulsive disorder: advances in diagnosis and treatment. *JAMA* 317 1358–1367. 10.1001/jama.2017.2200 28384832

[B37] Holtzheimer P. E. Husain M. M. Lisanby S. H. Taylor S. F. Whitworth L. A. Mcclintock S. (2017). Subcallosal cingulate deep brain stimulation for treatment-resistant depression: a multisite, randomised, sham-controlled trial. *Lancet Psychiatry* 4 839–849. 28988904 10.1016/S2215-0366(17)30371-1

[B38] Horn A. (2019). The impact of modern-day neuroimaging on the field of deep brain stimulation. *Curr. Opin. Neurol.* 32 511–520. 10.1097/wco.0000000000000679 30844863

[B39] Horn A. Blankenburg F. (2016). Toward a standardized structural-functional group connectome in MNI space. *Neuroimage* 124 310–322. 10.1016/j.neuroimage.2015.08.048 26327244

[B40] Horn A. Li N. Dembek T. A. Kappel A. Boulay C. Ewert S. (2019). Lead-DBS v2: towards a comprehensive pipeline for deep brain stimulation imaging. *Neuroimage* 184 293–316. 10.1016/j.neuroimage.2018.08.068 30179717 PMC6286150

[B41] Horn A. Reich M. Vorwerk J. Li N. Wenzel G. Fang Q. (2017). Connectivity predicts deep brain stimulation outcome in Parkinson disease. *Ann. Neurol.* 82 67–78. 10.1002/ana.24974 28586141 PMC5880678

[B42] Jakab A. Werner B. Piccirelli M. Kovacs K. Martin E. Thornton J. S. (2016). Feasibility of diffusion tractography for the reconstruction of intra-thalamic and cerebello-thalamic targets for functional neurosurgery: a multi-vendor pilot study in four subjects. *Front. Neuroanat.* 10:76. 10.3389/fnins.2015.00076 27462207 PMC4940380

[B43] Kessler R. C. (2012). The costs of depression. *Psychiatr. Clin. North Am.* 35 1–14. 22370487 10.1016/j.psc.2011.11.005 PMC3292769

[B44] Kessler R. C. Chiu W. T. Demler O. Merikangas K. R. Walters E. E. (2005). Prevalence, severity, and comorbidity of 12-month DSM-IV disorders in the national comorbidity survey replication. *Arch. Gen. Psychiatry* 62 617–627. 10.1001/archpsyc.62.6.617 15939839 PMC2847357

[B45] Kim S. J. Roh D. Jung H. H. Chang W. S. Kim C. H. Chang J. W. (2018). A study of novel bilateral thermal capsulotomy with focused ultrasound for treatment-refractory obsessive-compulsive disorder: 2-year follow-up. *J. Psychiatry Neurosci.* 43 327–337. 10.1503/jpn.170188 30125241 PMC6158029

[B46] Lehman J. F. Greenberg B. D. Mcintyre C. C. Rasmussen S. A. Haber S. N. (2011). Rules ventral prefrontal cortical axons use to reach their targets: implications for diffusion tensor imaging tractography and deep brain stimulation for psychiatric illness. *J. Neurosci.* 31 10392–10402. 10.1523/jneurosci.0595-11.2011 21753016 PMC3445013

[B47] Li N. Baldermann J. C. Kibleur A. Treu S. Akram H. Elias G. J. B. (2020). A unified connectomic target for deep brain stimulation in obsessive-compulsive disorder. *Nat. Commun.* 11:3364. 10.1038/s41467-020-16734-3 PMC7335093 32620886

[B48] Liebrand L. C. Caan M. W. A. Schuurman P. R. Van Den Munckhof P. Figee M. Denys D. (2019). Individual white matter bundle trajectories are associated with deep brain stimulation response in obsessive-compulsive disorder. *Brain Stimul.* 12 353–360. 10.1016/j.brs.2018.11.014 30522916

[B49] Lujan J. L. Chaturvedi A. Choi K. S. Holtzheimer P. E. Gross R. E. Mayberg H. S. (2013). Tractography-activation models applied to subcallosal cingulate deep brain stimulation. *Brain Stimul.* 6 737–739. 10.1016/j.brs.2013.03.008 23602025 PMC3772993

[B50] Luyten L. Hendrickx S. Raymaekers S. Gabriels L. Nuttin B. (2016). Electrical stimulation in the bed nucleus of the stria terminalis alleviates severe obsessive-compulsive disorder. *Mol. Psychiatry* 21 1272–1280. 10.1038/mp.2015.124 26303665

[B51] Makris N. Rathi Y. Mouradian P. Bonmassar G. Papadimitriou G. Ing W. I. (2016). Variability and anatomical specificity of the orbitofrontothalamic fibers of passage in the ventral capsule/ventral striatum (VC/VS): precision care for patient-specific tractography-guided targeting of deep brain stimulation (DBS) in obsessive compulsive disorder (OCD). *Brain Imag. Behav.* 10 1054–1067. 10.1007/s11682-015-9462-9 26518214 PMC4851930

[B52] Mallet L. Polosan M. Jaafari N. Baup N. Welter M. L. Fontaine D. (2008). Subthalamic nucleus stimulation in severe obsessive-compulsive disorder. *N. Engl. J. Med.* 359 2121–2134. 19005196 10.1056/NEJMoa0708514

[B53] Mayberg H. S. Lozano A. M. Voon V. Mcneely H. E. Seminowicz D. Hamani C. (2005). Deep brain stimulation for treatment-resistant depression. *Neuron* 45 651–660. 15748841 10.1016/j.neuron.2005.02.014

[B54] Mayberg H. S. Riva-Posse P. Crowell A. L. (2016). Deep brain stimulation for depression: keeping an eye on a moving target. *JAMA Psychiatry* 73 439–440. 10.1001/jamapsychiatry.2016.0173 27049731

[B55] Mendelsohn D. Lipsman N. Lozano A. M. Taira T. Bernstein M. (2013). The contemporary practice of psychiatric surgery: results from a global survey of functional neurosurgeons. *Stereotact. Funct. Neurosurg.* 91 306–313. 10.1159/000348323 23797416

[B56] Milad M. R. Pitman R. K. Ellis C. B. Gold A. L. Shin L. M. Lasko N. B. (2009). Neurobiological basis of failure to recall extinction memory in posttraumatic stress disorder. *Biol. Psychiatry* 66 1075–1082. 10.1016/j.biopsych.2009.06.026 19748076 PMC2787650

[B57] Nanda P. Banks G. P. Pathak Y. J. Sheth S. A. (2017). Connectivity-based parcellation of the anterior limb of the internal capsule. *Hum. Brain Mapp.* 38 6107–6117. 10.1002/hbm.23815 28913860 PMC6206867

[B58] Nuttin B. Cosyns P. Demeulemeester H. Gybels J. Meyerson B. (1999). Electrical stimulation in anterior limbs of internal capsules in patients with obsessive-compulsive disorder. *Lancet* 354:1526. 10.1016/s0140-6736(99)02376-4 10551504

[B59] Petersen M. V. Lund T. E. Sunde N. Frandsen J. Rosendal F. Juul N. (2017). Probabilistic versus deterministic tractography for delineation of the cortico-subthalamic hyperdirect pathway in patients with Parkinson disease selected for deep brain stimulation. *J. Neurosurg.* 126 1657–1668. 10.3171/2016.4.jns1624 27392264

[B60] Rabin J. S. Davidson B. Giacobbe P. Hamani C. Cohn M. Illes J. (2020). Neuromodulation for major depressive disorder: innovative measures to capture efficacy and outcomes. *Lancet Psychiatry* 29:S2215-0366(20)30187-5 [Epub ahead of print]. 10.1016/S2215-0366(20)30187-5 33129374

[B61] Ramasubbu R. Clark D. L. Golding S. Dobson K. S. Mackie A. Haffenden A. (2020). Long versus short pulse width subcallosal cingulate stimulation for treatment-resistant depression: a randomised, double-blind, crossover trial. *Lancet Psychiatry* 7 29–40. 10.1016/s2215-0366(19)30415-8 31860455

[B62] Rasmussen S. A. Noren G. Greenberg B. D. Marsland R. Mclaughlin N. C. Malloy P. J. (2018). Gamma ventral capsulotomy in intractable obsessive-compulsive disorder. *Biol. Psychiatry* 84 355–364. 10.1016/j.biopsych.2017.11.034 29361268

[B63] Raymaekers S. Luyten L. Bervoets C. Gabriels L. Nuttin B. (2017). Deep brain stimulation for treatment-resistant major depressive disorder: a comparison of two targets and long-term follow-up. *Transl. Psychiatry* 7:e1251. 10.1038/tp.2017.66 29087373 PMC5682606

[B64] Reznikov R. Bambico F. R. Diwan M. Raymond R. J. Nashed M. G. Nobrega J. N. (2018). Prefrontal cortex deep brain stimulation improves fear and anxiety-like behavior and reduces basolateral amygdala activity in a preclinical model of posttraumatic stress disorder. *Neuropsychopharmacology* 43 1099–1106. 10.1038/npp.2017.207 28862251 PMC5854795

[B65] Reznikov R. Binko M. Nobrega J. N. Hamani C. (2016). Deep brain stimulation in animal models of fear, anxiety, and posttraumatic stress disorder. *Neuropsychopharmacology* 41 2810–2817. 10.1038/npp.2016.34 26932819 PMC5061888

[B66] Reznikov R. Diwan M. Nobrega J. N. Hamani C. (2015). Towards a better preclinical model of PTSD: characterizing animals with weak extinction, maladaptive stress responses and low plasma corticosterone. *J. Psychiatr. Res.* 61 158–165. 10.1016/j.jpsychires.2014.12.017 25575638

[B67] Riva-Posse P. Choi K. S. Holtzheimer P. E. Crowell A. L. Garlow S. J. Rajendra J. K. (2018). A connectomic approach for subcallosal cingulate deep brain stimulation surgery: prospective targeting in treatment-resistant depression. *Mol. Psychiatry* 23 843–849. 10.1038/mp.2017.59 28397839 PMC5636645

[B68] Riva-Posse P. Choi K. S. Holtzheimer P. E. Mcintyre C. C. Gross R. E. Chaturvedi A. (2014). Defining critical white matter pathways mediating successful subcallosal cingulate deep brain stimulation for treatment-resistant depression. *Biol. Psychiatry* 76 963–969. 10.1016/j.biopsych.2014.03.029 24832866 PMC4487804

[B69] Riva-Posse P. Inman C. S. Choi K. S. Crowell A. L. Gross R. E. Hamann S. (2019). Autonomic arousal elicited by subcallosal cingulate stimulation is explained by white matter connectivity. *Brain Stimul.* 12 743–751. 10.1016/j.brs.2019.01.015 30738778

[B70] Sankar T. Chakravarty M. M. Jawa N. Li S. X. Giacobbe P. Kennedy S. H. (2020). Neuroanatomical predictors of response to subcallosal cingulate deep brain stimulation for treatment-resistant depression. *J. Psychiatry Neurosci.* 45 45–54. 10.1503/jpn.180207 31525860 PMC6919920

[B71] Schlaepfer T. E. Bewernick B. H. Kayser S. Madler B. Coenen V. A. (2013). Rapid effects of deep brain stimulation for treatment-resistant major depression. *Biol. Psychiatry* 73 1204–1212. 10.1016/j.biopsych.2013.01.034 23562618

[B72] Seminowicz D. A. Mayberg H. S. Mcintosh A. R. Goldapple K. Kennedy S. Segal Z. (2004). Limbic-frontal circuitry in major depression: a path modeling metanalysis. *Neuroimage* 22 409–418. 10.1016/j.neuroimage.2004.01.015 15110034

[B73] Talairach J. Hécaen H. David M. (1949). Lobotomie préfrontale limitée par électrocoagulation des fibres thalamo-frontales à leur émergence du bras antérieur de la capsule interne. *Rev. Neurol.* 83:59.

[B74] Trivedi M. H. Rush A. J. Wisniewski S. R. Nierenberg A. A. Warden D. Ritz L. (2006). Evaluation of outcomes with citalopram for depression using measurement-based care in STAR^∗^D: implications for clinical practice. *Am. J. Psychiatry* 163 28–40. 10.1176/appi.ajp.163.1.28 16390886

[B75] van der Wal J. M. Bergfeld I. O. Lok A. Mantione M. Figee M. Notten P. (2019). Long-term deep brain stimulation of the ventral anterior limb of the internal capsule for treatment-resistant depression. *J. Neurol. Neurosurg. Psychiatry* 91 189–195. 10.1136/jnnp-2019-321758 31801845 PMC6996094

[B76] Van Essen D. C. Smith S. M. Barch D. M. Behrens T. E. Yacoub E. Ugurbil K. (2013). The WU-Minn Human Connectome Project: an overview. *Neuroimage* 80 62–79. 10.1016/j.neuroimage.2013.05.041 23684880 PMC3724347

[B77] Widge A. S. Deckersbach T. Eskandar E. N. Dougherty D. D. (2016). Deep brain stimulation for treatment-resistant psychiatric illnesses: what has gone wrong and what should we do Next? *Biol. Psychiatry* 79 e9–e10. 26212895 10.1016/j.biopsych.2015.06.005

[B78] Widge A. S. Malone D. A. Jr. Dougherty D. D. (2018). Closing the loop on deep brain stimulation for treatment-resistant depression. *Front. Neurosci.* 12:175. 10.3389/fnins.2015.00075 29618967 PMC5871707

[B79] Winter L. Saryyeva A. Schwabe K. Heissler H. E. Runge J. Alam M. (2020). Long-term deep brain stimulation in treatment-resistant obsessive-compulsive disorder: outcome and quality of life at four to eight years follow-up. *Neuromodulation* 15 [Epub ahead of print]. 10.1111/ner.13232 32667114

[B80] World Health Organization (2017). *Depression and Other Common Mental Disorders: Global Health Estimates.* Geneva: WHO.

